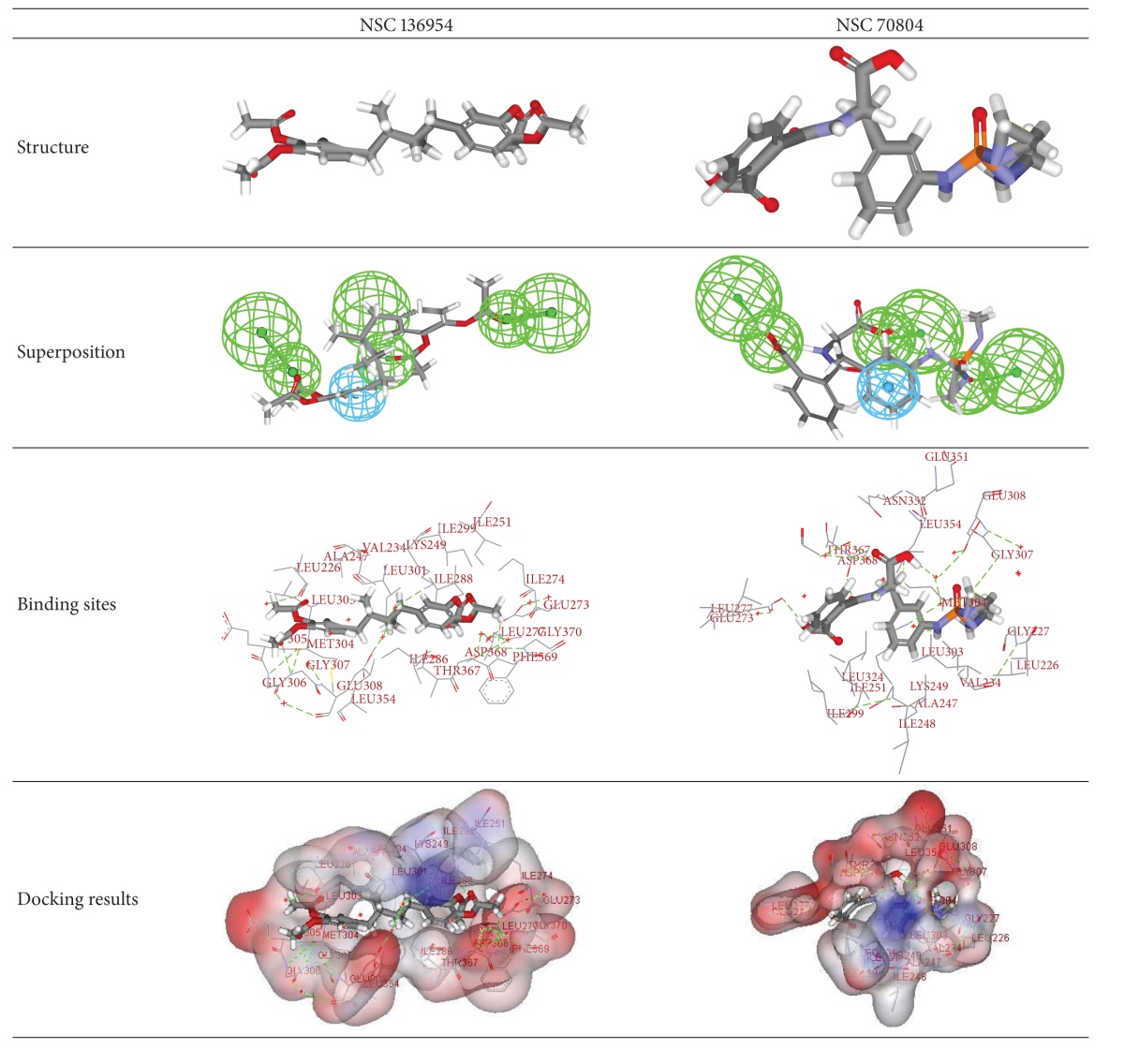# Novel Design Strategy for Checkpoint Kinase 2 Inhibitors Using Pharmacophore Modeling, Combinatorial Fusion, and Virtual Screening

**DOI:** 10.1155/2014/359494

**Published:** 2014-04-23

**Authors:** Chun-Yuan Lin, Yen-Ling Wang

**Affiliations:** ^1^Department of Computer Science and Information Engineering, Chang Gung University, Taoyuan 33302, Taiwan; ^2^Research Center for Emerging Viral Infections, Chang Gung University, Taoyuan 33302, Taiwan

## Abstract

Checkpoint kinase 2 (Chk2) has a great effect on DNA-damage and plays an important role in response to DNA double-strand breaks and related lesions. In this study, we will concentrate on Chk2 and the purpose is to find the potential inhibitors by the pharmacophore hypotheses (PhModels), combinatorial fusion, and virtual screening techniques. Applying combinatorial fusion into PhModels and virtual screening techniques is a novel design strategy for drug design. We used combinatorial fusion to analyze the prediction results and then obtained the best correlation coefficient of the testing set (*r*_test_) with the value 0.816 by combining the Best_train_Best_test_ and Fast_train_Fast_test_ prediction results. The potential inhibitors were selected from NCI database by screening according to Best_train_Best_test_ + Fast_train_Fast_test_ prediction results and molecular docking with CDOCKER docking program. Finally, the selected compounds have high interaction energy between a ligand and a receptor. Through these approaches, 23 potential inhibitors for Chk2 are retrieved for further study.

## 1. Introduction

DNA-damage is induced by ionizing radiation, genotoxic chemicals, or collapsed replication forks, and when DNA was damaged or the responses of cells were failure, the mutation associated with the breast or ovarian cancer of genes may occur. To prevent and repair the DNA-damage, mammalian cells will control and stabilize the genome by cell cycle checkpoint. The checkpoint pathway consists of several kinases, such as ataxia telangiectasia mutated protein (ATM [1, 2]), ataxia telangiectasia and Rad3-related protein (ATR [1, 2]), checkpoint kinase 1 (Chk1 [3, 4]), and checkpoint kinase 2 (Chk2 [5–8]). ATM and ATR are upstream kinases passing messages to downstream kinases and phosphorylating several proteins that initiate the activation of the DNA-damage checkpoint. Moreover, ATM is a primarily pathway to activate p53 (protein 53 [9]) by Chk2, and ATR may influence the phosphorylation of Chk1. Both Chk1 and Chk2 are key components in DNA-damage; however, their cellular activities are different. Chk1 is involved in S and G2 phases of the cell cycle with ATR pathway. By contrast, Chk2 is activated in all phases through ATM-dependent pathway and plays an important role in response to DNA double-strand breaks and related lesions. Furthermore, Chk1 is an unstable protein and lacks the forkhead-associated domain (FHA) which was involved in several processes that protect against cancer and can be found in Chk2. Therefore, we concentrate on Chk2 in this study.

Chk2 is a protein containing 543 amino acid residues and the structure of Chk2 consists of some functional elements, including the N-terminal SQ/TQ cluster domain (SCD), FHA, and the N-terminal serine/threonine kinase domain (KD) [5–8]. The SCD is known to be the preferred site with the residue Thr68 for phosphorylation to respond to DNA-damage by ATM/ATP kinases. The FHA domain is a phosphopeptide recognition domain found in many regulatory proteins and thought to bind to the phosphoThr68 segment of SCD [5–8, 10–14]. Hence it is a good candidate for interactions of Chk2 with its upstream regulators or downstream targets in the cell-cycle-checkpoint signaling. The KD occupies almost the entire carboxy-terminal half of Chk2 and has been identified based on their homology with serine/threonine kinases. Some studies reported that when DNA was damaged, Chk2 is activated by ATM/ATR through the phosphorylation of residue Thr68. Moreover, Chk2 induces transautophosphorylation of residues Thr383 and Thr387 and then cis-phosphorylation of residue Ser516 [5–8, 10–14]. After that, Chk2 will phosphorylate several downstream substrates, such as BRCA1 (breast cancer 1, early onset [15, 16]), Cdc25A (cell division cycle 25 homolog A), Cdc25C, and p53 [7, 8, 10]. Several researches indicated that Chk2 phosphorylates Cdc25A which is considered an oncogene on the residue Ser123 in S phase of cell cycle, and it also phosphorylates Cdc25C on the residue Ser216 in G2 phase helping prevent mitotic entry in cells with damaged DNA [5]. Furthermore, BRCA1 and p53 are involved in DNA repair process in the breast or ovarian cancer. BRCA1 is a human caretaker gene and helps repair damaged DNA or destroys cells which cannot be repaired. The p53 is a tumor suppressor protein involved in preventing cancer in human and plays an important role in the G1 checkpoint in response to DNA damaging agents. We consider that the sites of the phosphorylations are important in the drug design for cell survival when DNA is damaged.

**Figure 1:**
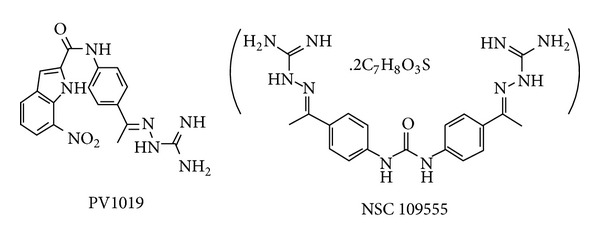
Two-dimensional chemical structures of known Chk2 inhibitors. The experimental IC_50_ of PV1019 and NSC 109555 were 138 nM and 240 nM, respectively.

Recently, several studies identified the inhibitors of Chk2 [6–8, 10–14], and they also showed the crystal structures of Chk2 complex, such as PDB: 1GXC, 2W7X, and, and so forth. They are selective, reversible, and ATP-competitive Chk2 inhibitors demonstrating that they effectively restrain the radiation-induced phosphorylation of Chk2. In addition, several selective Chk2 inhibitors have been also identified (two examples were shown in Figure 1) and the researches indicated that they are potential and selective inhibitors of Chk2 with chemotherapeutic and radiosensitization potential. On structure-based drug design, several developments of Chk2 were published [17, 18]. Quantitative structure-activity relationship model (QSAR model) is a regression or classification model and is an important technique in the rational drug design. It is used to correlate the structure properties of compounds with their biological activities. The method to predict the quality by QSAR was improved by considering the three-dimensional structure QSAR (3D-QSAR) [19–24] of targeted inhibitor. Therefore, the compound structure can be directly optimized in the 3D space. The comparative molecular field analyses (CoMFA) [18, 25–30] and the comparative molecular similarity indices analyses (CoMSIA) [18, 27–32] for Chk2 inhibitors were performed by ligand-based and receptor-guided alignment. They used the cocrystal structure from protein data bank (PDB code: 2CN8) [7], and then they identified new plausible binding modes used as template for 3D-QSAR [18]. There is another research of Chk2 studied in QSAR/QSPR [17] providing structures that will improve reducing the side effects of Chk2 inhibitors.

Pharmacophore [20–24, 33–35] is a set of structural features responsible for the biological activity of a molecule. It allowed compounds with diverse structures to find the common chemical features by ligand pharmacophore mapping, and that is different from CoMFA and CoMSIA with the common structure constraint. Thus, pharmacophore can explain how diverse ligands bind to a receptor site by these features and visualize the feature of potential chemical interactions between ligands and receptors. Moreover, pharmacophore can easily and quickly identify candidate inhibitors for a target protein based on 3D query. Therefore, in this work, we first used 3D-QSAR study to build pharmacophore hypotheses (denoted as PhModels) for Chk2 inhibitors by HypoGen Best, Fast, and Caesar algorithms, respectively. Then we used the combinatorial fusion to select and combine prediction results for improving the predictive accuracy in biological activities of inhibitors. Virtual screening is a computational technique used in drug discovery research. There are two categories of screening techniques: ligand-based and structure-based. In this work, for ligand-based virtual screening, we used the selected PhModels as 3D structure query by pharmacophore hypothesis screening that each compound in National Cancer Institute (NCI) database will be mapped onto the pharmacophoric features of selected PhModels. When the chemical features of a compound fit the generated PhModels, it will be selected. All of feasible compounds in NCI database were selected in this work. Finally, the potential inhibitors were retrieved from selected compounds by using molecular docking program to predict the conformation and interaction energy between Chk2 and ligand. Applying combinatorial fusion into PhModels and virtual screening techniques is a novel design strategy for drug design and can help medicinal chemists to identify or design new Chk2 inhibitors. Besides, the potential inhibitors of Chk2 retrieved in this work can be estimated by biologists for further study.

## 2. Materials and Methods

### 2.1. Biological Data Collection

In order to construct the PhModels, at first, we collected the Chk2 inhibitors with two-dimensional structures and the biological activity values from the ChEMBL database [36]. Then, according to the structure variations and chemical differences in the kinase inhibitor activity, 158 known Chk2 inhibitors were selected and retrieved. The biological activity of 158 known Chk2 inhibitors was represented as IC_50_ (nanomolar, nM). There are 260,071 compounds from the NCI database (release version 3, http://cactus.nci.nih.gov/download/nci/) which were used in the database screening and molecular docking approach in this work.

### 2.2. Training and Testing Sets Selection

**Figure 2:**
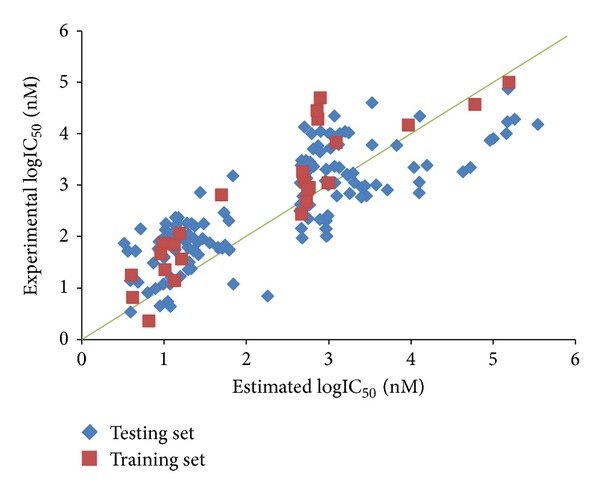
The scatter diagram of training set and testing set inhibitors.

Before generating PhModels, we should divide the 158 Chk2 inhibitors into the training set and testing set, respectively. The rules used to select training set inhibitors are according to the following requirements as suggested by the Accelrys Discovery Studio. (1) All selected inhibitors should have clear and concise information including structure features and activity range. (2) At a minimum, 16 diverse inhibitors for training set were selected to ensure the statistical significance. (3) The training set should contain the most and the least active inhibitors. (4) The biological activities of the inhibitors spanned at least 4 orders of magnitude. Based on the above four rules, the 158 Chk2 inhibitors were divided, and the scatter diagram of training set and testing set inhibitors was shown in Figure 2. Figure 2 demonstrates the distribution of the inhibitors in the training set and testing set, and the representative points of the testing set are close to those of the training set. The training set with 25 inhibitors is used to construct PhModels, and the IC_50_ values of these 25 inhibitors are ranged from 2.3 to 100,000 nM (Table 1). The testing set with remaining 133 inhibitors is used to test the predictive ability of generated PhModels, and the IC_50_ values of the 133 testing set inhibitors are ranged from 3.4 to 74,000 nM (Table 2). After selecting the training set and testing set inhibitors, we established PhModels at first, and then we used the correlation analysis to estimate the prediction abilities of PhModels.

### 2.3. Pharmacophore Generation

**Figure 3:**
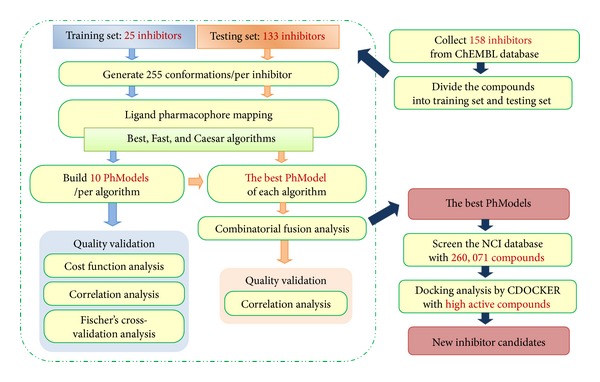
The workflow of PhModel generation for Chk2 inhibitors.

The workflow of PhModel generation for Chk2 inhibitors was shown in Figure 3. In this study, we used the HypoGen program [37] in Accelrys Discovery Studio 2.1 to generate PhModels. At the initial step, 3D conformations of the training set inhibitors were generated by using “3D-QSAR Pharmacophore Generation protocol” with the Best, Fast, and Caesar generating algorithms, respectively, based on the CHARMm-like force field. The conformational-space energy was constrained ≤20 kcal/mol which represented the maximum allowed energy above the global minimum energy. For each training set inhibitor, the number of the diverse 3D conformations was set to ≤255. All other parameters were set as default values. Following the above rules, the 3D conformations were generated, and then we can construct the PhModel by using “Ligand Pharmacophore Mapping protocol.” Each of the ten PhModels using HypoGen Best, Fast, and Caesar algorithms were generated in this study.

### 2.4. Combinatorial Fusion

In this study, we use a combinatorial fusion technique to facilitate prediction results selection and combination for improving predictive accuracy in biological activities of inhibitors. The combinatorial fusion we take is analogous to that used in information retrieval [38, 39], pattern recognition [40], molecular similarity searching and structure-based screening [41], and microarray gene expression analysis [42]. These works have demonstrated the following remark [43].

**Remark 1**

For a set of multiple scoring systems, each with a score function and a rank function, we have that (a) the combination of multiple scoring systems would improve the prediction accuracy only if (1) each of the systems has a relatively high performance, and (2) the individual systems are distinctive (or diversified), and (b) rank combination performs better than score combination under certain conditions.

Given an inhibitor and for each prediction result *A*, let *s*_*A*_ be a function as the predicted biological activity and it is represented as a real number. We view the function *s*_*A*_ as the score function. Since *s*_*A*_ only assigns a number not a set of numbers, in this work, no rank function would be used for an inhibitor. Therefore, the rank combination and the rule (b) in Remark 1 are not considered in the study. Suppose we have *m* prediction results (*m* scoring functions). There are combinatorially 2^*m*^ − 1 combinations for all *m* individual prediction results $\left(\sum_{k=1}^{m}\left(\begin{array}{c} m\\ k \end{array}\right)=2^{m}-1\right)$ with score functions. The total number of combinations to be considered for predicting biological activity of an inhibitor is 2^*m*^ − 1. This number of combinations can become huge when the number of prediction results *m* is large. Moreover, we have to evaluate the predictive power of each combination across all inhibitors. This study would start with combining only two prediction results which still retain fairly good prediction power.

Suppose *m* prediction results *A*_*i*_, *i* = 1,2,…, *m*, are given with score function *s*_*Ai*_; there are several different ways of combination. Among others, there are score combination, voting, linear average combination, and weighted combination [38–42]. Voting is computationally simple and better than simple linear combinations when applied to the situation with large number of prediction results. However, a better alternative is to reduce the number of prediction results to a smaller number and then these prediction results are combined. In this paper, we reduce the set of prediction results to those which perform relatively well and then use the rank/score function to decide whether to combine by score. In this paper, we use the rules (a) (1) and (a) (2) stated in Remark 1 as our guiding principle to select prediction results and to decide on the method of combination. After generating each of the ten PhModels by using HypoGen Best, Fast, and Caesar algorithms for training set inhibitors, each of the best PhModel (denoted as Best_train_, Fast_train_, and Casear_train_) was evaluated by its correlation coefficient of the training set (*r*_train_). Then these best PhModels were used to predict the biological activities of testing set inhibitors by using HypoGen Best, Fast, and Caesar algorithms. Therefore, there are nine prediction results (denoted as *Z*_train_ × *Z*_test_, *Z* = {Best, Fast, Caesar}, that is, Best_train_Best_test_) generated for testing set inhibitors. Using data fusion, results from various prediction results are combined to obtain predictions with larger accuracy rate. The diversity rank/score function is used to select the most suitable prediction results for combination. If these three best PhModels were selected, there are nine prediction results and then there are 2^9^ − 1 = 511 combinations. According to the rule (a) (1) in Remark 1, the *r*_train_ of Casear_train_ is far less than those of Best_train_ and Fast_train_ (Table 1); then, the Casear_train_ was not considered in the combinations. Therefore, there are six prediction results (*Z*_1train_ × *Z*_2test_, *Z*_1_ = {Best, Fast} and *Z*_2_ = {Best, Fast, Caesar}) and 2^6^ − 1 = 63 combinations. A special diversity rank/score graph was used to choose the best discriminating prediction results for further combination.

For an inhibitor *p*_*i*_ in the testing set *P* = {*p*_1_, *p*_2_,…, *p*_*t*_} and the pair of prediction results *A* and *B*, the diversity score function *d*_*i*_(*A*, *B*) is defined as *d*_*i*_(*A*, *B*) = ∑|*s*_*A*_ − *s*_*B*_|. When there are *q* prediction results selected (in this study, *q* = 6), there are $\left(\begin{array}{c} q\\ 2 \end{array}\right)=q(q-1)/2$ (in this study, the number is 15) diversity score functions. If we let *i* vary and fix the prediction result pair (*A*, *B*), then *d*_*i*_(*A*, *B*) is the diversity score function *s*_(*A*,*B*)_ from *P* = {*p*_1_, *p*_2_,…, *p*_*t*_}. Sorting *s*_(*A*,*B*)_ into descending order would lead to the diversity rank function *r*_(*A*,*B*)_. Consequently, the diversity rank/score function *f*_(*A*,*B*)_ is defined as *f*_(*A*,*B*)_ = (*s*_(*A*,*B*)_∘*r*_(*A*,*B*)_^−1^)(*j*) = *s*_(*A*,*B*)_(*r*_(*A*,*B*)_^−1^(*j*)), where *j* is in *T* = {1, 2, 3,…, *t*}. We note that the set *T* is different from the set *P* which is the testing set considered. The set *T* is used as the index set for the diversity rank function value and |*T*| = *t* is indeed the cardinality of *P*. The diversity rank/score function *f*_(*A*,*B*)_ so defined exhibits the diversity trend of the prediction result pair (*A*, *B*) across the whole spectrum of input set of *t* inhibitors and is independent of the specific inhibitor under study. For two prediction results *A* and *B*, the graph of the diversity rank/score function*f*_(*A*,*B*)_(*j*) is called the diversity rank/score graph. This study aims to examine all the *q*(*q* − 1)/2 diversity rank/score graphs to see which pair of prediction results would give the larger diversity measurement according to the rule (a) (2) in Remark 1.

### 2.5. Database Screen

After examining 15 diversity rank/score graphs, the PhModels *A* and *B* determined from the best prediction result pair were used to screen the NCI database for new Chk2 inhibitor candidates. Under the PhModel, pharmacophore hypothesis screening can be used to screen small molecule database to retrieve the compounds as potential inhibitors that fit the pharmacophoric features. In this study, the “Search 3D Database protocol” with the Best/Fast/Casear Search option in Accelrys Discovery Studio 2.1 was employed to search the NCI database with 260,071 compounds. We could filter out and select the compounds in the NCI database based on the estimated activity and chemical features of PhModel.

### 2.6. Molecular Docking

**Figure 4:**
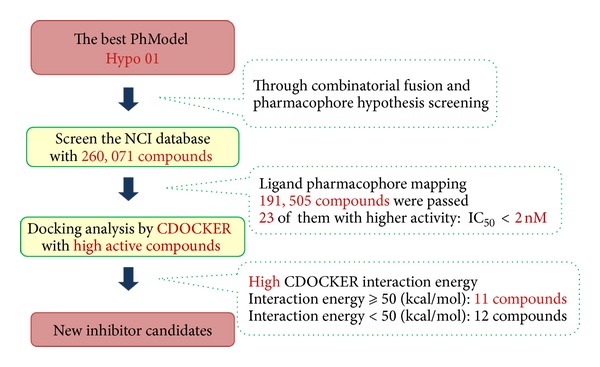
The workflow of database screening and molecular docking approach for new Chk2 inhibitor candidates.

After the database screening approach, the selected compounds can be further estimated according to the interaction energy between a receptor and a ligand through the molecular docking approach. In this study, selected compounds in the NCI database were docked into Chk2 active sites by CDOCKER docking program, and then their CDOCKER interaction energies were estimated. Finally, new potential candidates were retrieved from the NCI database with high interaction energy. The workflow of database screening and molecular docking approach was shown in Figure 4.

## 3. Results

### 3.1. PhModel Generation Results

Each of the ten PhModels using 25 training set inhibitors and HypoGen Best, Fast, and Caesar algorithms was generated by selecting hydrogen bond acceptor (A), hydrogen bond donor (D), and hydrophobic (H) and hydrophobic aromatic (HYAR) features. Each of the best PhModels, Best_train_, Fast_train_, and Casear_train_, was evaluated with the best *r*_train_, and the predicted biological activities of training set inhibitors and *r*_train_ were listed in Table 1, respectively. From Table 1, the Best_train_ obtained better *r*_train_ of value 0.955 than those by Fast_train_ and Casear_train_. Moreover, the *r*_train_ of Casear_train_ is far less than those of Best_train_ and Fast_train_. Hence, HypoGen Best algorithm was used individually to generate the PhModels for most of target genes in the past. According to rule (a) (1) in Remark 1, the Casear_train_ was not considered to be used for the prediction of testing set inhibitors.

### 3.2. Correlation Analysis of Testing Set Inhibitors

The testing set inhibitors were predicted by Best_train_ and Fast_train_ with HypoGen Best, Fast, and Caesar algorithms. Therefore, there are six prediction results, Best_train_Best_test_ (denoted as BB), Best_train_Fast_test_ (denoted as BF), Best_train_Casear_test_ (denoted as BC), Fasr_train_Best_test_ (denoted as FB), Fast_train_Fast_test_ (denoted as FF), and Fast_train_Casear_test_ (denoted as FC), for testing set inhibitors. The predicted biological activities of testing set inhibitors and *r*_test_ by these six prediction results were listed in Table 2, respectively. From Table 2, for the Best_train_, the best *r*_test_ of value 0.81 was achieved by the Best_train_Best_test_; for the Fast_train_, the best *r*_test_ of value 0.728 was achieved by the Fast_train_Fast_test_. However, the Best_train_Best_test_ obtained the best *r*_test_ in overall; moreover, the prediction results in the Best_train_ all outperform those in the Fast_train_.

### 3.3. Combinatorial Fusion Analysis

**Figure 5:**
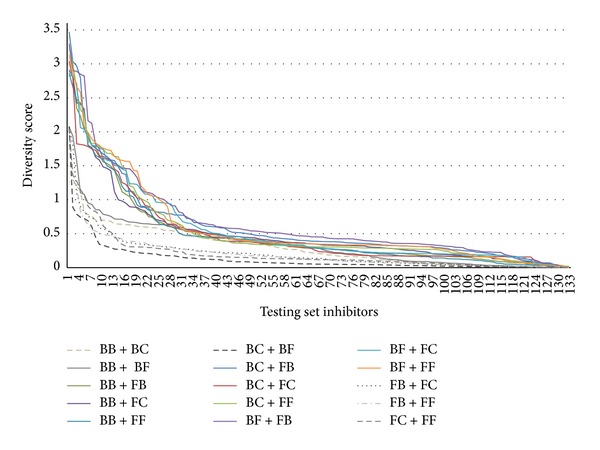
The diversity rank/score graphs for 15 combinations of prediction results.

**Figure 6:**
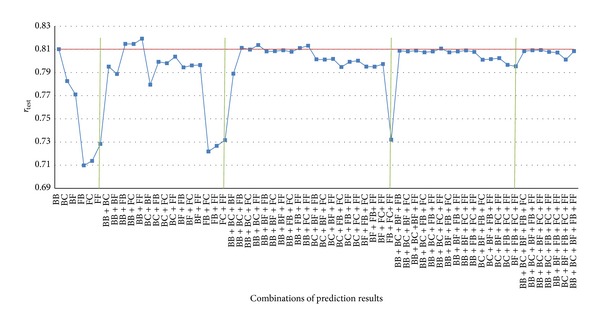
The *r*_test_ for all of 63 combinations from six prediction results.

Under the six prediction results, the diversity score function *d*_*i*_(*A*, *B*) was calculated for each testing set inhibitor by a pair of prediction results (*A*, *B*). There are 15 diversity score functions *s*_(*A*,*B*)_ that were performed at first and then these diversity score functions were sorted to become the diversity rank function *r*_(*A*,*B*)_, respectively. Finally, 15 diversity rank/score functions *f*_(*A*,*B*)_ were represented as diversity rank/score graphs shown in Figure 5. Among 15 diversity rank/score graphs, there are several combinations (gray color) that have less diversity scores than those by others, such as BB + BC, BB + BF, and FB + FB, shown in Figure 5. It means that these combinations may have less *r*_test_ than those by others according to rule (a) (2) in Remark 1. In other words, several combinations, such as BB + FC (purple color), BB + FF (blue color), and BF + FF (orange color), may have larger *r*_test_ than those by others due to larger diversity scores. For the six prediction results, all of the 63 combinations were preformed and evaluated by its *r*_test_, respectively, as shown in Figure 6. In Figure 6, for 15 pairs of two prediction results, the combinations BB + FB, BB + FC, and BB + FF have larger *r*_test_ than those by others. Moreover, the combination BB + FF has best *r*_test_ of value 0.816 among 15 combinations, even for 63 combinations. Besides, the average *r*_test_ by the combinations is larger than the individual prediction results. It means that the predictive accuracy for Chk2 inhibitors may be improved by considering the Best_train_ and Fast_train_ concurrently.

### 3.4. Database Screen Results

The best PhModels, Best_train_ and Fast_train_, were used to screen the NCI database with 260,071 compounds for new Chk2 inhibitor candidates by using HypoGen Best and Fast algorithms, respectively. The Best_train_Best_test_ and Fast_train_Fast_test_ prediction results for NCI database were combined in order to filter out possible false positive candidates. Of the 260,071 compounds, 191,505 passed the screening and best fitted to the chemical features in 3D space. 23 drug-like compounds that had an estimated IC_50_ value of less than 2 nM were studied in a molecular docking study (Figure 4).

### 3.5. Molecular Docking Results

23 drug-like compounds along with the training set compounds were docked into the active sites that were defined based on the bound inhibitor, PV1019, in a crystal structure of Chk2 (PDB: 2W7X). We used CDOCKER program to confirm that inhibitor candidates bind to the receptor. CDOCKER uses molecular dynamics (MD) in conjunction with the CHARMm force field to individually dock the compounds into the binding sites. The coordinates of Chk2 from the Chk2/PV1019 crystal structure were used after removing PV1019 and solvent molecules and adding protein hydrogen atoms. After docking each screened compound, its interaction energy value was calculated. The PV1019 was redocked into the Chk2 binding site by the CDOCKER program. Its-CDOCKER interaction energy was calculated by CDOCKER and determined to be 37.786 (kal/mol). The 23 drug-like compounds were docked into the Chk2 binding sites. Finally, there are 21 drug-like compounds with CDOCKER interaction energies greater than 37.786 (kal/mol). In addition, 11 drug-like compounds had high interaction value greater than 50 (kal/mol) (Figure 4) and the top 2 are NSC136954 with 61.239 (kal/mol) and NSC70804 with 58.967 (kal/mol), respectively, kept for future characterization as inhibitors. The 21 drug-like compounds with their estimated IC_50_ values and CDOCKER interaction energy greater than 37.786 (kal/mol) were shown in Table 3.

The structures and characteristics of the top 2 compounds are given in Table 4, and we can find that some active site residues were identified from the Chk2 complex. The interaction sites of NSC136954 were Leu226, Val234, Ala247, Lys249, Ile251, Glu273, Ile274, Leu277, Ile286, Ile288, Ile299, Leu301, Leu303, Met304, Glu305, Gly306, Gly307, Glu308, Leu354, Thr367, Asp368, Phe369, and Gly370. On the other hand, the interaction sites of NSC70804 were Leu226, Leu227, Val234, Ala247, Ile248, Lys249, Ile251, Glu273, Leu277, Ile299, Leu301, Leu303, Met304, Gly307, Glu308, Glu351, Asn352, Leu354, Thr367, and Asp368. Several studies indicated that they are involved in hydrophobic interactions with Val234, Ile251, Leu354, Ile299, and the aliphatic portions of the side chains of Lys249, Thr367, and Asp368, in addition to several interactions of van der Waals or hydrophobic with Leu226, Val234, Leu303, Gly307, Leu354, and the aliphatic portions the side chains of Met304 and Glu308 [10, 11]. Furthermore, the quinazoline was sandwiched between the lipophilic side chains of Val234 and Leu354, with the side chains of Ala247, Leu301, and Leu303 also contributing to a hydrophobic surface surrounding the core and an interaction between the pyrazole and Lys249 is likely to account for the increase in Chk2 potency [12]. And residue Thr367 of Chk2 is a serine in Chk1. Portions of the glycine-rich P-loop in Chk2, which is located directly above the inhibitor, are disordered (residues 229–231), whereas this loop is well defined in the structure of Chk1, and Leu301 in Chk2 corresponds to the “gatekeeper” residue in many kinases, which has been found to form contacts with bound inhibitors and is poorly conserved [44].

## 4. Conclusions

In this study, a novel design strategy for drug design was proposed to apply combinatorial fusion into PhModels and virtual screening techniques. 158 Chk2 inhibitors were divided into the training set and testing set, respectively. For 25 training set inhibitors, three best PhModels, Best_train_, Fast_train_, and Casear_train_, were generated at first, and then six prediction results for 133 testing set inhibitors were used for calculating 15 diversity rank/score functions. Finally, the combination Best_train_Best_test_ and Fast_train_Fast_test_ prediction results achieved the best *r*_test_ of value 0.816 among 63 combinations. Through these approaches, 23 potential Chk2 inhibitors with IC_50_ value less than 2 nM and interaction energy value larger than 37.786 (kal/mol) are retrieved from NCI database. This study can help medicinal chemists to identify or design new Chk2 inhibitors. Besides, the potential inhibitors of Chk2 retrieved in this work can be estimated by biologists for further study.

## Figures and Tables

**Table 1:** Experimental and estimated IC_50_ values of training set inhibitors.

CHEMBL ID	Experimental IC_50_ (nM)	Estimated IC_50 _(nM)		
		Best_train_	Fast_train_	Caesar_train_
CHEMBL195041	2.3	15	9.9	1129
CHEMBL193990	6.6	6.8	6.2	942
CHEMBL248935	14	20	20	833
CHEMBL195320	18	8.5	6.2	942
CHEMBL176164	23	19	23	1151
CHEMBL250765	37	30	22	950
CHEMBL362677	47	23	23	1153
CHEMBL249959	70	110	20	1000
CHEMBL250992	72	47	6.9	72
CHEMBL251155	110	220	23	756
CHEMBL588536	270	670	790	78578
CHEMBL400772	470	2200	268	231
CHEMBL367390	640	2000	2237	1028
CHEMBL608262	830	1200	1456	94262
CHEMBL401105	900	1000	235	20
CHEMBL176115	1100	970	1044	1449
CHEMBL253542	1200	1100	189	3.8
CHEMBL592490	1800	860	1275	93360
CHEMBL589090	6700	1700	1419	3561
CHEMBL199299	15000	22000	233	1745
CHEMBL251629	19000	3600	615	411
CHEMBL259084	28000	6800	31827	5300
CHEMBL251628	37000	63000	1360	24786
CHEMBL438485	50000	16000	320	243
CHEMBL589501	100000	160000	48276	96926

Correlation coefficient** **(*r*_train_)		0.955	0.840	0.238

**Table 2:** Experimental and estimated IC_50_ values of testing set inhibitors.

CHEMBL ID	Experimental IC_50_ (nM)	Estimated IC_50 _(nM)						
		Best_train_			Fast_train_			Best_train_Best_test_ + Fast_train_Fast_test_
		Best_test_	Fast_test_	Caesar_test_	Best_test_	Fast_test_	Caesar_test_	
CHEMBL195177	3.4	3.9	5.2	5.1	14.8	6.2	12.3	6.2
CHEMBL359881	4.4	12.0	46.1	42.3	17.3	22.5	29.4	22.5
CHEMBL179717	4.5	9.0	42.1	43.2	14.3	21.9	29.3	21.9
CHEMBL175553	5.5	11.2	57.8	43.1	17.6	20.5	29.1	20.5
CHEMBL192161	7	183.4	74.6	36.7	258.7	253.9	291.4	253.9
CHEMBL191969	8.2	6.4	15.3	14.3	10.5	9.4	13.5	9.4
CHEMBL175472	9.8	7.9	48.4	9.9	12.4	22.5	29.1	22.5
CHEMBL361378	12	9.9	48.1	43.7	14.7	21.7	29.3	21.7
CHEMBL362255	12	11.9	55.4	42.3	15.5	22.9	29.4	22.9
CHEMBL369254	12	70.2	51.3	45.6	38.0	23.7	31.5	23.7
CHEMBL364978	13	4.8	4.9	5.1	13.5	6.5	12.3	6.5
CHEMBL195846	14	3.9	5.0	5.1	12.8	6.2	12.3	6.2
CHEMBL179583	16	12.2	48.5	43.1	13.4	23.2	28.9	23.2
CHEMBL178972	17	15.9	52.5	43.1	18.0	23.3	29.1	23.3
CHEMBL250360	23	19.8	46.8	43.7	17.6	21.7	29.4	21.7
CHEMBL175879	24	21.6	57.0	42.3	24.0	23.2	29.2	23.2
CHEMBL179267	31	7.4	63.5	42.8	20.5	21.2	29.4	21.2
CHEMBL192022	32	20.3	47.5	42.6	23.9	22.4	29.4	22.4
CHEMBL250158	39	10.1	20.4	43.7	19.1	21.5	29.3	21.5
CHEMBL363339	41	10.1	59.8	42.2	18.1	24.0	29.5	24.0
CHEMBL250555	45	26.3	48.2	42.9	24.5	22.7	29.4	22.7
CHEMBL250359	52	3.7	5.2	3.5	20.6	7.0	28.8	7.0
CHEMBL251585	52	4.5	3.0	3.1	11.9	4.9	10.7	4.9
CHEMBL398529	53	23.0	48.4	43.5	21.4	22.4	29.4	22.4
CHEMBL178971	55	62.4	44.9	43.1	41.7	22.1	29.3	22.1
CHEMBL427879	55	13.7	45.4	42.4	16.9	19.9	29.5	19.9
CHEMBL250963	57	8.6	44.1	42.9	17.8	21.5	27.2	21.5
CHEMBL251170	60	51.3	45.3	43.1	26.3	21.5	29.4	21.5
CHEMBL250759	61	45.6	47.4	43.3	36.6	23.2	29.5	23.2
CHEMBL367263	61	19.7	50.3	9.6	17.3	23.9	29.1	23.9
CHEMBL250159	67	55.7	45.3	43.1	25.7	17.2	29.4	17.2
CHEMBL398467	70	11.4	46.5	42.7	21.0	20.4	29.4	20.4
CHEMBL250796	73	3.3	3.4	4.2	14.7	6.0	14.4	6.0
CHEMBL250957	74	36.1	44.9	43.7	30.5	20.4	29.5	20.4
CHEMBL206609	77	25.9	51.0	44.0	9.6	16.3	17.8	16.3
CHEMBL400755	78	8.8	26.6	43.0	12.9	22.5	29.4	22.5
CHEMBL249569	80	11.1	48.3	43.3	17.0	22.9	28.0	22.9
CHEMBL193397	81	9.9	43.8	42.8	14.5	21.2	28.4	21.2
CHEMBL438868	82	18.9	42.0	43.1	13.1	19.0	29.3	19.0
CHEMBL249566	86	17.6	48.8	43.1	23.5	22.8	29.6	22.8
CHEMBL249345	90	23.2	47.6	43.0	20.9	20.0	25.0	20.0
CHEMBL399146	90	29.8	47.9	42.7	28.4	22.1	29.3	22.1
CHEMBL602931	92	483.6	560.1	506.8	645.9	594.0	588.2	594.0
CHEMBL249347	95	10.1	46.3	43.1	27.2	20.1	29.2	20.1
CHEMBL193476	100	951.1	914.5	925.8	2435.2	1027.4	1981.9	1027.4
CHEMBL250361	100	14.2	6.6	14.5	14.9	20.9	29.6	20.9
CHEMBL248934	109	20.0	52.5	43.3	14.0	22.4	29.4	22.4
CHEMBL249750	110	12.7	49.3	43.5	20.7	21.6	29.3	21.6
CHEMBL208463	133	10.4	46.0	41.0	968.6	2768.3	2670.7	2768.3
CHEMBL250566	140	5.2	17.3	26.3	18.4	20.9	29.6	20.9
CHEMBL251256	140	936.4	2453.5	2240.6	192.6	215.1	215.5	215.1
CHEMBL437331	142	471.8	521.6	450.6	64.2	222.1	60.7	222.1
CHEMBL249541	157	23.8	42.5	43.0	16.0	18.8	29.3	18.8
CHEMBL249776	158	13.9	47.2	43.7	16.7	20.9	29.4	20.9
CHEMBL249350	174	30.6	51.2	43.4	22.5	23.3	29.4	23.3
CHEMBL249546	176	10.6	47.3	42.8	17.9	21.0	29.4	21.0
CHEMBL251364	176	21.7	43.9	43.1	17.8	19.7	29.1	19.7
CHEMBL399933	180	14.8	45.0	43.5	20.8	18.9	29.4	18.9
CHEMBL400287	180	19.3	45.5	43.5	21.5	19.8	29.4	19.8
CHEMBL175780	200	61.7	47.5	42.3	36.0	21.9	29.5	21.9
CHEMBL176326	200	935.6	926.3	913.6	1896.0	986.2	1980.7	986.2
CHEMBL590335	210	791.5	721.8	807.4	616.3	639.0	644.7	639.0
CHEMBL398561	220	575.1	926.2	571.5	209.1	278.3	287.9	278.3
CHEMBL249777	231	15.0	45.7	42.5	25.5	21.7	29.4	21.7
CHEMBL442282	233	13.9	46.9	42.3	21.5	22.5	29.4	22.5
CHEMBL195599	250	981.5	908.0	925.8	2266.7	1057.6	1981.9	1057.6
CHEMBL176015	290	54.1	52.8	54.4	24.0	29.3	28.0	29.3
CHEMBL251284	310	484.8	429.7	533.7	189.6	196.8	217.4	196.8
CHEMBL600441	310	454.6	559.9	516.5	300.5	513.5	254.1	513.5
CHEMBL599581	410	594.7	496.6	509.1	506.1	252.9	299.2	252.9
CHEMBL592784	420	462.2	492.0	475.1	216.7	203.8	198.3	203.8
CHEMBL1197465	580	2492.8	8163.9	5925.9	995.1	896.8	488.9	896.8
CHEMBL590809	600	492.4	539.0	534.3	816.4	536.2	549.3	536.2
CHEMBL1197456	610	2871.8	7537.6	6733.2	615.7	4139.2	3514.6	4139.2
CHEMBL590637	610	1251.9	1786.7	1121.1	2379.0	1650.9	1262.3	1650.9
CHEMBL591518	680	1797.9	1804.0	1516.5	6075.0	4714.4	2726.5	4714.4
CHEMBL598973	700	12585.6	151896.0	84151.4	1047.3	396.5	1318.1	396.5
CHEMBL251368	710	27.7	45.9	43.8	13.4	21.2	29.1	21.2
CHEMBL1197303	800	5153.6	36481.1	6594.4	1277.8	681.9	4077.9	681.9
CHEMBL1197320	890	2537.8	7191.1	6002.7	1559.4	517.5	420.0	517.5
CHEMBL1197528	960	2752.7	7737.1	5925.9	765.7	654.9	559.0	654.9
CHEMBL215803	1000	3760.6	140257.0	74847.0	9672.7	50053.5	49263.9	50053.5
CHEMBL253324	1000	996.2	2416.5	603.1	264.7	553.1	272.4	553.1
CHEMBL589347	1100	458.3	560.2	482.3	209.7	299.1	196.7	299.1
CHEMBL604784	1100	1188.4	1365.6	1205.7	2305.3	1962.3	1307.9	1962.3
CHEMBL1197529	1120	2047.4	11678.5	8090.0	3368.8	3648.8	3659.9	3648.8
CHEMBL1197326	1130	12645.5	45428.8	7432.7	1138.2	465.0	416.0	465.0
CHEMBL176041	1200	925.0	906.8	913.6	1933.6	1040.0	1980.7	1040.0
CHEMBL590079	1350	548.9	548.5	550.6	1000.9	860.3	855.1	860.3
CHEMBL605083	1400	489.2	613.8	554.4	1224.9	1401.0	1345.6	1401.0
CHEMBL175481	1500	69.2	57.7	49.2	1853.9	1526.1	1852.1	1526.1
CHEMBL205906	1540	1696.1	1109.3	1153.0	980.6	17655.5	937.5	17655.5
CHEMBL590808	1600	1662.5	1799.1	1709.4	51882.2	48813.2	48302.3	48813.2
CHEMBL1170748	1700	1974.2	1940.4	1652.8	3251.4	2734.9	407.6	2734.9
CHEMBL253541	1800	43066.9	42831.1	79622.8	1255.9	1099.1	15566.3	1099.1
CHEMBL176554	1900	572.3	7226.3	9126.2	1096.1	3352.6	3926.3	3352.6
CHEMBL377597	2000	938.5	6719.7	5644.6	50.4	116.9	105.4	116.9
CHEMBL1170749	2200	10874.0	30873.8	3995.6	5835.1	25044.3	1090.5	25044.3
CHEMBL590336	2200	507.8	594.0	516.4	568.9	544.3	521.1	544.3
CHEMBL590807	2200	52944.6	70480.3	52639.4	44898.4	49196.7	36195.2	49196.7
CHEMBL600868	2200	1342.8	1603.8	1312.9	7373.6	5797.7	4864.3	5797.7
CHEMBL398759	2300	666.0	2847.2	1010.2	447.2	924.3	309.0	924.3
CHEMBL604459	2300	1176.9	2299.2	1369.3	614.2	877.8	682.5	877.8
CHEMBL179383	2400	15724.9	14621.7	14257.4	4508.5	4132.8	4023.9	4132.8
CHEMBL592489	2400	469.2	494.6	486.9	204.7	240.5	208.4	240.5
CHEMBL425904	2800	605.0	651.0	534.9	478.1	451.9	488.6	451.9
CHEMBL150894	3000	537.8	844.9	797.6	186.8	600.2	289.0	600.2
CHEMBL590793	3000	475.4	2480.4	1251.5	192.0	251.7	205.4	251.7
CHEMBL600865	4400	796.9	2072.3	1223.3	198.1	261.3	228.1	261.3
CHEMBL249253	5000	659.8	503.6	499.5	406.6	227.9	309.5	227.9
CHEMBL587506	5200	1050.8	1293.3	1083.2	2201.3	2162.7	1656.0	2162.7
CHEMBL204930	5800	755.5	1260.7	1105.5	47881.6	47912.3	47868.0	47912.3
CHEMBL554900	5900	6722.8	580526.0	807309.0	745.4	428.2	47897.1	428.2
CHEMBL176276	6000	3360.2	8955.9	8476.6	2075.6	2054.0	1970.7	2054.0
CHEMBL589091	6100	1319.5	1308.4	1172.0	579.2	603.1	600.9	603.1
CHEMBL559781	7400	91783.7	822917.0	1230010.0	414.5	6865.5	47911.9	6865.5
CHEMBL249252	8000	100671.0	38586.8	31129.8	590.5	491.6	467.9	491.6
CHEMBL589089	9800	1070.7	1321.5	1002.7	38589.3	48136.2	41143.2	48136.2
CHEMBL217090	10000	628.8	1010.9	1548.5	390.6	451.8	910.1	451.8
CHEMBL217092	10000	1030.2	1199.3	1908.8	379.9	625.6	707.2	625.6
CHEMBL382588	10000	1365.2	5880.3	5488.7	2560.7	3993.4	3649.4	3993.4
CHEMBL590581	10000	145206.0	149922.0	108067.0	50609.5	49348.5	48302.3	49348.5
CHEMBL242753	10300	1742.5	3393.1	1941.1	1202.5	2613.0	1297.3	2613.0
CHEMBL398758	11000	1582.8	187965.0	1573.6	237.0	4333.9	369.0	4333.9
CHEMBL399151	11000	812.1	1263.5	2069.9	334.5	1193.9	1271.6	1193.9
CHEMBL395080	13450	513.5	484.0	458.6	198.5	188.9	183.3	188.9
CHEMBL1171533	15000	349604.0	296515.0	159368.0	26784.5	26699.3	45506.4	26699.3
CHEMBL602729	17000	148756.0	149052.0	139465.0	224.1	324.7	229.4	324.7
CHEMBL249255	19000	182486.0	42615.8	41578.3	1698.2	616.1	683.9	616.1
CHEMBL202930	21730	12828.4	11945.6	12145.8	213.3	219.7	217.6	219.7
CHEMBL589986	22000	1167.1	1337.4	1143.5	53747.5	49155.6	49316.8	49155.6
CHEMBL251471	40000	3358.9	1946.2	2075.7	512.2	423.7	3427.4	423.7
CHEMBL560056	74000	152006.0	156723.0	208466.0	223.2	208.1	190.4	208.1

Correlation coefficient (*r*_test_)		0.810	0.771	0.783	0.710	0.728	0.714	**0.816**

**Table 3:** The 21 drug-like compounds with their estimated IC_50 _values and CDOCKER interaction energy greater than 37.786 (kal/mol).

Name	Estimated IC_50 _(nM)	Interaction energy (kal/mol)
**NSC 136954**	**1.989**	**61.239**
**NSC 70804**	**1.682**	**58.967**
NSC 158029	1.885	57.944
NSC 603427	1.87	56.963
NSC 57782	1.6855	56.54
NSC 16739	1.5385	56.342
NSC 720227	1.914	55.839
NSC 618702	1.862	55.196
NSC 195178	1.7015	51.351
NSC 653142	1.557	51.19
NSC 653143	1.577	50.055
NSC 32200	1.901	49.439
NSC 342015	1.6515	47.327
NSC 343685	1.7615	46.436
NSC 205750	1.875	45.542
NSC 96538	1.705	44.344
NSC 210455	1.7935	42.258
NSC 314654	1.947	42.082
NSC 179894	1.6135	41.707
NSC 91710	1.701	40.533
NSC 370907	1.8785	40.502

**Table 4:** The structures and characteristics of the top 2 compounds.